# The Role of Situational Leadership on Job Satisfaction, Organizational Citizenship Behavior (OCB), and Employee Performance

**DOI:** 10.3389/fpsyg.2022.896539

**Published:** 2022-05-09

**Authors:** Sahala Benny Pasaribu, Francisca Sestri Goestjahjanti, Srinita Srinita, Dewiana Novitasari, Budi Haryanto

**Affiliations:** ^1^Faculty of Economics and Business, Trilogi University, Jakarta, Indonesia; ^2^Sekolah Tinggi Ilmu Ekonomi Insan Pembangunan, Tangerang, Indonesia; ^3^Faculty of Economics and Business, Syiah Kuala University, Banda Aceh, Indonesia

**Keywords:** situational leadership, job satisfaction, employee performance, OCB, SMEs

## Introduction

According to Desky et al. (2020) and Kadiyono et al. (2020), performance in an organization is carried out by all human resources in the organization, both the leader and employees. Each employee has ability based on their knowledge, skills, and competencies. According to Mustofa and Muafi (2021) and Nugroho et al. (2020a) each employee has ability based on their knowledge, skills, and competencies. However, employees also have personalities, attitudes, and behaviors that can affect their performance. According to Asbari et al. (2021), performance is work (output) quality and quantity achieved by human resources including punctuality in carrying out their work tasks in accordance with the responsibilities assigned to them. Human resources are the assets of a company to achieve success (Fahlevi, 2021). According to Mustafa et al. (2021), it takes a leader with the right leadership style to direct all existing human resources in the company to achieve company goals. The role of leadership is important and needed to harmonize various needs and create a conducive work situation. Because small and medium enterprises (SMEs) face intense competition, SME leaders must pay close attention to employee behavior that will support their performance (Indrawan et al., 2020).

According to Nugroho et al. (2020a,b) and Nadeak et al. (2021), positive employee behavior will be able to support individual performance and organizational performance for better organizational development. Furthermore, according to Robbins and Judge (2015, p. 40), organizations with employees who have good organizational citizenship behavior (OCB) will have better performance than other organizations. Apart from the leadership and OCB factors that are closely related to employee performance, employee satisfaction is also very influential on their performance at work (Nugroho et al., 2020a). Research results from Purwanto et al. (2021) and Ridlwan et al. (2021) show that leadership style affects performance through job satisfaction. This means that if the leadership style is good, then job satisfaction and performance will increase/improve. On the other hand, if the leadership style is not good, job satisfaction and employee performance will be lower/less good.

Discussion on job satisfaction cannot be separated from the factors that can affect employee job satisfaction. Further, according to Nugroho et al. (2020b) and Nadeak et al. (2021), performance is the result of certain work on activities over a certain period of time. Therefore, performance is a result achieved by a person according to the responsibilities applicable to the job in question. Therefore, performance is not only about the personal characteristics shown by a person but also the results of work that has been and will be done by someone. Employee performance in SMEs has not yet achieved the company's targeted expectations. Employee job satisfaction as one of the influencing factors still cannot be met, so the leader should pay attention to aspects of communication, opportunities, work environment, and others related to employee job satisfaction in order to further improve employee performance.

## Method

The research method employed in this study was quantitative using a survey. In this study, the respondents were SME employees totaling 200 people who were determined through a simple random sampling method. The data collection method was performed through the distribution of online questionnaires to respondents through social media. Data analysis in this study used the path analysis method or Path Analysis using SmartPLS software (SMEs in Banten, Indonesia).

**Figure 1:**
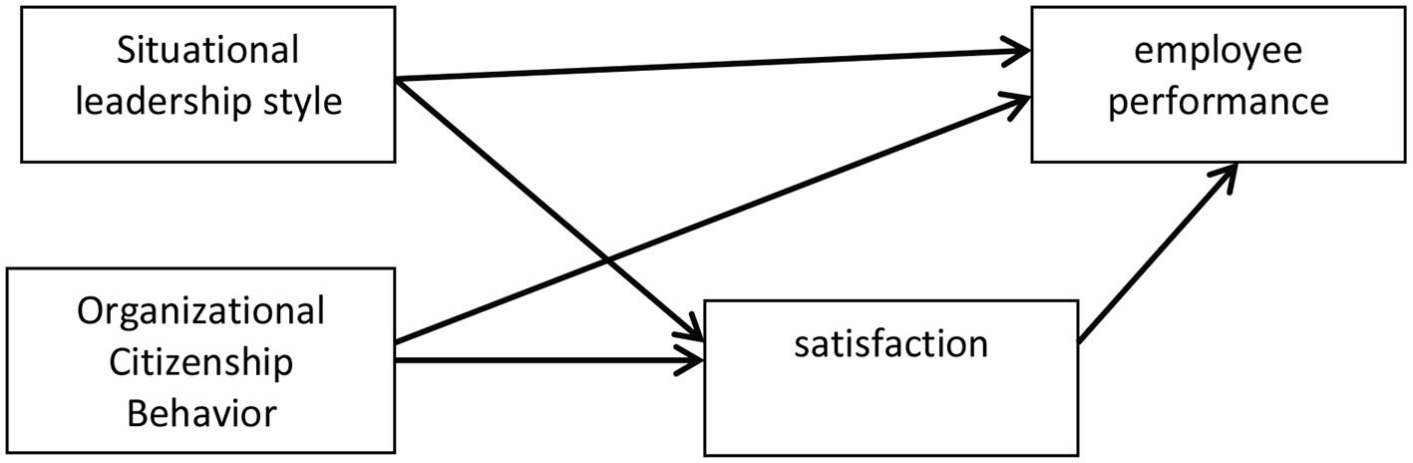
Research method.

According to Figure 1 the hypotheses of this study are:

- H1: Situational leadership style has a positive effect on job satisfaction.
- H2: Situational leadership style has a positive effect on employee performance.
- H3: Job satisfaction has a positive effect on employee performance.
- H4: OCB has a positive effect on employee performance.
- H5: OCB has a positive effect on job satisfaction.

## Results and Discussion

Based on data analysis, the results of research on SME employees can be described as follows, situational leadership style has a positive effect on job satisfaction in SMEs as seen from the coefficient value of 0.610, indicating that situational leadership style has a positive effect of 61% on job satisfaction. The value of the *t* statistic of 6.124, which is >1.96, and the *p*-value of 0.000 which is smaller than 0.05, indicates that the situational leadership style has a significant effect on employee job satisfaction in SMEs. Situational leadership style has a positive effect on employee performance in SMEs as seen from the coefficient value of 0.504, indicating that situational leadership style has a positive effect of 50.4% on employee performance. The value of the *t* statistic of 4.231 which is >1.96 and the *p*-value of 0.000 which is smaller than 0.05, indicates that the situational leadership style has a significant effect on employee performance in SMEs. Job satisfaction has a positive effect on employee performance in SMEs as seen from the coefficient value of 0.609 which indicates that job satisfaction has a positive effect of 60.9% on employee performance. The value of the *t* statistic of 4.817, which is >1.96, and the *p*-value of 0.001 which is smaller than 0.05, indicates that job satisfaction has a significant effect on employee performance in SMEs. OCB has a positive effect on employee performance in SMEs as seen from the coefficient value of 0.409, indicating that OCB has a positive effect of 40.9% on employee performance. The value of the *t* statistic of 9.098, which is >1.96, and the *p*-value of 0.001, which is smaller than 0.05, indicates that OCB has a significant effect on employee performance in SMEs.

Situational leadership style has a positive effect on job satisfaction and employee performance in SMEs as seen from the coefficient value of 0.509, indicating that situational leadership style has a positive effect of 50.9% on job satisfaction and employee performance. The value of the *t* statistic of 3.90, which is >1.96, and the *p*-value of 0.000, which is smaller than 0.05, indicates that the situational leadership style has a significant effect on job satisfaction and employee performance in SMEs. OCB has a positive effect on job satisfaction and employee performance in SMEs as seen from the coefficient value of 0.509, indicating that OCB has a positive effect of 50.9% on job satisfaction and employee performance. The value of the *t* statistic of 7.09 which is >1.96 and the *p*-value of 0.001 which is smaller than 0.05, indicates that OCB has a positive effect on job satisfaction and employee performance in SMEs.

Research data analysis shows that situational leadership style has a positive and significant effect on job satisfaction and employee performance. According to Purwanto et al. (2022) and Nugroho et al. (2020b), the leadership style has provided two-way directions and easy-to-understand explanations for employees. Besides, employees are also involved in decision-making and providing ideas so that they feel they have a stake in company activities, which affects their satisfaction. However, this is not optimal, and employees have not been able to improve their performance as a result. The results of this study indicate that employees have the freedom to use their skills at work. This is what encourages employees to take on extra work to continue prioritizing the company's interests and taking responsibility for the tasks assigned to them. As a result, this study demonstrates that situational leadership style has a positive and significant effect on OCB. Previous studies conducted by Sunarsi et al. (2020) and Vizano et al. (2020) show that servant leadership has a positive and significant relationship and effect on organizational commitment and OCB. Therefore, it shows that this study supports previous research. Then, the results of observations through the distribution of questionnaires in the field reveal that job satisfaction has a negative and insignificant effect on performance. This is inseparable from the condition of SMEs as private companies. Of course, employees have sufficient salaries to meet their daily needs. Therefore, everything they have received so far, including salaries and awards, is considered an award from the company. As a result, their job satisfaction can encourage increased performance. This study is contrary to the findings of a study conducted by Sunarsi et al. (2020) which in relation to satisfaction with performance suggests that when employee satisfaction changes, this change will bring changes to employee performance.

The OCB has a positive and significant effect on employee performance. This is supported by the level of awareness of each employee in carrying out their duties, which will improve their performance. In addition, employee awareness of the need to complete their work within the planned timeframe encourages them to work harder. The results of this study are in accordance with the opinions of Vizano et al. (2020) and Wuryani et al. (2021), which suggest that organizations that have employees with good OCB, will have better performance than other organizations.

## Conclusion

Based on the results of the analysis obtained, the conclusions from this study are as follows: Situational leadership style has a significant positive effect on employee job satisfaction. Therefore, the better a leader applies situational leadership to employees, the greater the satisfaction of SME employees. Situational leadership style has a significant positive effect on the OCB of employees. Therefore, a good leader's ability to apply a situational leadership style will further increase the engagement of SME employees. Job satisfaction has a negative and insignificant effect on employee performance. Therefore, decreasing employee job satisfaction does not always increase the performance of SMEs. OCB has a significant positive effect on employee performance. As a result, the higher the OCB of employees, the higher the performance of SME employees. Situational leadership style has a significant positive effect on employee performance mediated by job satisfaction. Therefore, good situational leadership does not always improve employee performance through SME employee job satisfaction. Situational leadership style has a significant positive effect on employee performance mediated by OCB. As a result, good situational leadership will increase employee OCB so that it has an impact on increasing employee performance in SMEs.

## Author Contributions

SP contributed to conceptualization, methodology, investigation, curation, analysis, funding acquisition, and writing. FG and SS helped in the investigation, curation, analysis, and writing. DN and BH helped in review, analysis, and writing. All authors contributed to the article and approved the submitted version.

## Conflict of Interest

The authors declare that the research was conducted in the absence of any commercial or financial relationships that could be construed as a potential conflict of interest.

## Publisher's Note

All claims expressed in this article are solely those of the authors and do not necessarily represent those of their affiliated organizations, or those of the publisher, the editors and the reviewers. Any product that may be evaluated in this article, or claim that may be made by its manufacturer, is not guaranteed or endorsed by the publisher.
